# Intra-observer and inter-observer repeatability of ocular surface interferometer in measuring lipid layer thickness

**DOI:** 10.1186/s12886-015-0036-9

**Published:** 2015-05-15

**Authors:** Yang Zhao, Carin Lay San Tan, Louis Tong

**Affiliations:** Yong Loo Lin School of Medicine, National University of Singapore, Singapore, Singapore; Singapore National Eye Center, 11 Third Hospital Avenue, 168751 Singapore, Singapore; Ocular Surface Research Group, Singapore Eye Research Institute, 168751 Singapore, Singapore; Department of Cornea and External Eye Disease, Singapore National Eye Center, 11 Third Hospital Avenue, 168751 Singapore, Singapore; Office of Clinical, Academic and Faculty Affairs, Duke-NUS Graduate Medical School, Singapore, Singapore; Department of Ophthalmology, Yong Loo Lin School of Medicine, National University of Singapore, Singapore, Singapore

**Keywords:** Imaging, Human, Clinical study, Cornea, Lipid, Tear

## Abstract

**Background:**

Tear lipid morphology is important for normal tear function. Recently, there have been clinical studies using interferometry to assess lipid layer thickness (LLT). The aim of the study is to examine the repeatability of a commercially available interferometer.

**Methods:**

Two observers measured LLT in twenty Asian subjects (20 eyes) using an interferometer (LipiView® ocular surface interferometer, TearScience Inc, Morrisville, NC). Dry eye symptoms, tear break up time (TBUT) and corneal fluorescein staining were also prospectively evaluated.

**Results:**

Data for 20 participants are presented for either right or left eye (randomly selected). The mean LLT ± standard deviation of these participants was 53.53 ± 14.59 nm. When a single observer repeated the imaging on the same day, the coefficient of repeatability was 16 nm and the 95 % limits of agreement were between −11 nm and 18 nm. When a different observer repeated the scan, the coefficient of repeatability was 13 nm and limits of agreement were −9 nm and 16 nm. LLT was not significantly associated with TBUT, presence of any corneal staining in any corneal zones, or symptomatic status.

**Conclusion:**

With the repeatability of measurements being known, the significance of LLT changes measured by this interferometer may be better interpreted. In this small Asian study, the LLT was lower than previously reported studies.

## Background

Dry eye is a common condition that carries significant patient morbidity and healthcare cost [1, 2]. For many years, symptomatic dry eye has been qualitatively evaluated and cannot be externally graded in research trials. While there are routine quantitative tests, such as the tear break up time (TBUT) and Schirmer’s test, these tests are highly variable in their measurements [3]. Recently, advances have been made in developing more objective and reliable tests, which employ modalities such as optical coherence tomography [4–7], tear osmolarity measurement [8] and interferometry [9, 10].

Disturbance to the preocular tear film is a key feature of dry eye [11]. The preocular tear film, about 3 μm thick [12], provides vital nutrients to the corneal epithelium [13, 14], and serves as a barrier against the external environment [13, 15]. Being the first refractive interface for incident light, the tear film also plays an important role in ensuring good visual quality [16]. The tear lipid layer, measuring 20–180 nm in thickness [9, 14, 17–19], is the outermost layer of the tear film, superficial to the aqueous layer and the mucin layer.

The lipid layer has traditionally been thought to contribute to tear film stability [20–22]. Since the lipid layer serves as a barrier for the underlying aqueous tear to escape, it may reduce tear evaporation [23–25]. Blinking of the eyelids also plays an important role in the normal function and physiology of the ocular surface, including the reconstitution of the tear film [14, 26–29]. During each blink, the tear lipid layer dynamically changes in morphology [14, 27, 30]. Therefore, apart from the function of tear stability, the lipid layer thickness (LLT) is also a measure of firstly the regularity of the surface [31], secondly the evenness/dynamics of tear spreading [18, 32], and lastly the amount of underlying aqueous [22, 32].

Measurement of the LLT is potentially important in diseases of the ocular surface. The tear lipids are produced by the meibomian glands and a common ocular surface disease is meibomian gland dysfunction (MGD), defined as a chronic eyelid condition with occlusion of terminal meibomian gland ducts and qualitative and quantitative changes of the expressed meibum [33]. In hypersecretory MGD, LLT may be increased whereas in hyposecretory MGD, it may be reduced [34].

In addition, LLT is correlated to the number of expressible meibomian glands [35] and meibomian gland loss [19]. Measurement of LLT therefore leads to a greater understanding of diseases that affect lipid expression and aid in their assessment, such as in the diagnosis of MGD [36–39]. Some studies have also shown an increase in LLT after treatment of MGD [40, 41], suggesting that it may be used as a monitoring tool after commencement of treatment.

Despite the potential applications of LLT, it is challenging to directly quantify. The development of interferometric methods has made LLT assessment more feasible. Interferometry has received major scientific attention recently, partly related to technological advancement in imaging and publication of treatment trials [40, 41].

In interferometry, when white light is projected over the cornea, a color interference pattern is produced due to specular reflection at the lipid-aqueous interface [18]. By correlating interference color with LLT [18, 42], a recently released interferometer (LipiView® ocular surface interferometer, TearScience Inc, Morrisville, NC) can objectively quantify the LLT [9, 10]. Being the first commercial interferometer to do so, it can measure LLT in interferometric color unit, which is equivalent to nanometer. This is potentially more useful than evaluating LLT in ordinal grades [20, 43] and may be better for longitudinal evaluation of patients.

Repeatability of measurements is crucial in ensuring the reliability of results, but there is no existing data on the repeatability of this interferometer. There were also no studies on the repeatability of LLT in repeat scans. To address these issues, we aim to investigate the inter-observer and intra-observer repeatability of the LipiView® ocular surface interferometer in the measurement of LLT.

## Methods

### Participants

The SingHealth Centralised Institutional Review Board approved this study and it adhered to the tenets of the Declaration of Helsinki. This study was registered under the clinicaltrials.gov database (NCT01933165). 20 participants (20 eyes) were recruited from the public via poster recruitment and verbal announcement.

The inclusion criterion was the absence of prior dry eye diagnosis. Exclusion criteria were: eye surgery done within the past 3 months, and active ocular surface conditions such as infection or pterygium that may affect tear film stability. As dry eye is a heterogenous condition, one expects in clinical studies that groups of patients with varying disease severity and tear parameters are included. We do not expect that the studied interferometer will only be used for a specific type of dry eye patients. For this reason, the participant selection criteria were not excessively restrictive and aimed to include a variety of normal and mild dry eye cases.

Potential participants were screened for eligibility and written informed consent was sought for each participant by the investigators. Biodata, history of past contact lens wear, and history of ophthalmic surgery were documented.

### Symptom score

A dry eye questionnaire used in our previous study [44] was administered to each participant prior to the measurement of the lipid layer thickness. Participant was considered as symptomatic if any of the symptoms was reported as “often” or “all the time”.

### Interferometric assessment of lipid layer thickness

Each eye was assessed thrice by each of the two investigators (ZY and CTLS) using an interferometer (LipiView® ocular surface interferometer, TearScience Inc, Morrisville, NC). Both investigators were trained and validated for the use of the device. Between every measurement, there was a 5-min interval for participant to rest, during which time the participant removed his head from the chin rest. All measurements for each participant were performed on the same visit, in the same room with relatively unchanged conditions, namely room humidity, temperature and ambient lighting (clinic lighting).

For each measurement, the participant was instructed to rest his head on the chin-rest and to blink freely during imaging. The measurement area was digitally set over the cornea, about 1 mm above the inferior tear meniscus and manually focused with interface controls. The interferometer was run for its maximum recording duration and the recorded video was automatically analysed for LLT in nanometers based on recorded interferometric color units. The output LLTs were copied to the data-recording sheet and later collated for further analysis.

### Tear break up time and corneal fluorescein staining

Clinical evaluation of the eye was performed under slit lamp microscopy only after interferometry had been completed, as clinical evaluation may disrupt the lipid layer. The ocular surface was stained with fluorescein by introducing a wetted Fluoret® (1 mg Fluorescein Sodium Ophthalmic Strip, Bausch & Lomb, Rochester, NY) into the inferior fornix and the participant was instructed to blink afterward. Then, tear break-up time (TBUT) was measured once by recording the time taken for any dry spot to form over the tear film from the moment of eye opening [44]. A shorter TBUT indicates a less stable tear film and is associated with dry eyes. Afterwards, punctate staining or erosions of the corneal epithelium were documented and graded according to the Cornea and Contact Lens Research Unit (CCLRU) scheme as published [45]. Briefly, each of the five corneal zones was scored between 0 (no staining/scarring) to 4 (severe staining). The presence of clinically relevant staining in each corneal zone was taken as a CCLRU staining grade of 1 or greater.

### Visual acuity screening and comfort post imaging

Participants were screened for their best corrected spectacle visual acuity using a Snellen chart. Participants were asked about any ocular or periocular symptoms after the assessment.

### Statistical analysis

Data was tested for normality using the skewness and kurtosis test, Kolmogorov-Smirnov test**,** histogram and q-q plot. Coefficient of repeatability was calculated as 2 times the standard deviation of the differences [46]. Bland-Altman plots [46] were also plotted to assess both intra-observer and inter-observer repeatability and outliers were identified visually with scatter plots and boxplots. Linear regression was used for univariate and multivariate analysis of LLT. Correlation of LLT with TBUT was measured using Spearman’s rank correlation coefficient. Statistically significant difference was based on alpha of 0.05. All analyses were performed with SPSS, version 21 (SPSS Inc, Chicago, IL).

## Results

### Characteristics of participants

20 volunteers (20 eyes) were recruited for the study. We present the eye data from a randomized side (using a random number generator) for each patient. Table 1 shows the study sample’s charateristics, namely biodata, clinical history and parameters.

**Table 1:** Descriptive characteristics of participants

	Selected eyes (n = 20)
Age (years old) (mean, s.d.)	30.75 (12.89)
Gender	9 Male (45 %)
Race	19 Chinese (95 %), 1 Indian (5%)
Symptomatic status for dry eye	13 (65 %)
Past ocular surgery	2 (10 %)
(1 bilateral LASIK, 1 bilateral strabismus surgery)	
Current contact lens wear	2 (10 %)
Tear break-up time (s) (median, minimum, maximum)	3 (2, 7)
Tear break-up time <5 s	14 (70 %)
Presence of any corneal staining	8 (40 %)
Superior	0 (0 %)
Inferior	6 (30 %)
Nasal	1 (5 %)
Temporal	2 (10 %)
Central	3 (15 %)
Lipid layer thickness (nm) (mean, s.d.)	
Investigator 1:	56 (17)
Investigator 2:	52 (13)
Combined:	54 (15)

### Intra-observer and Inter-observer repeatability of LLT

**Fig. 1:**
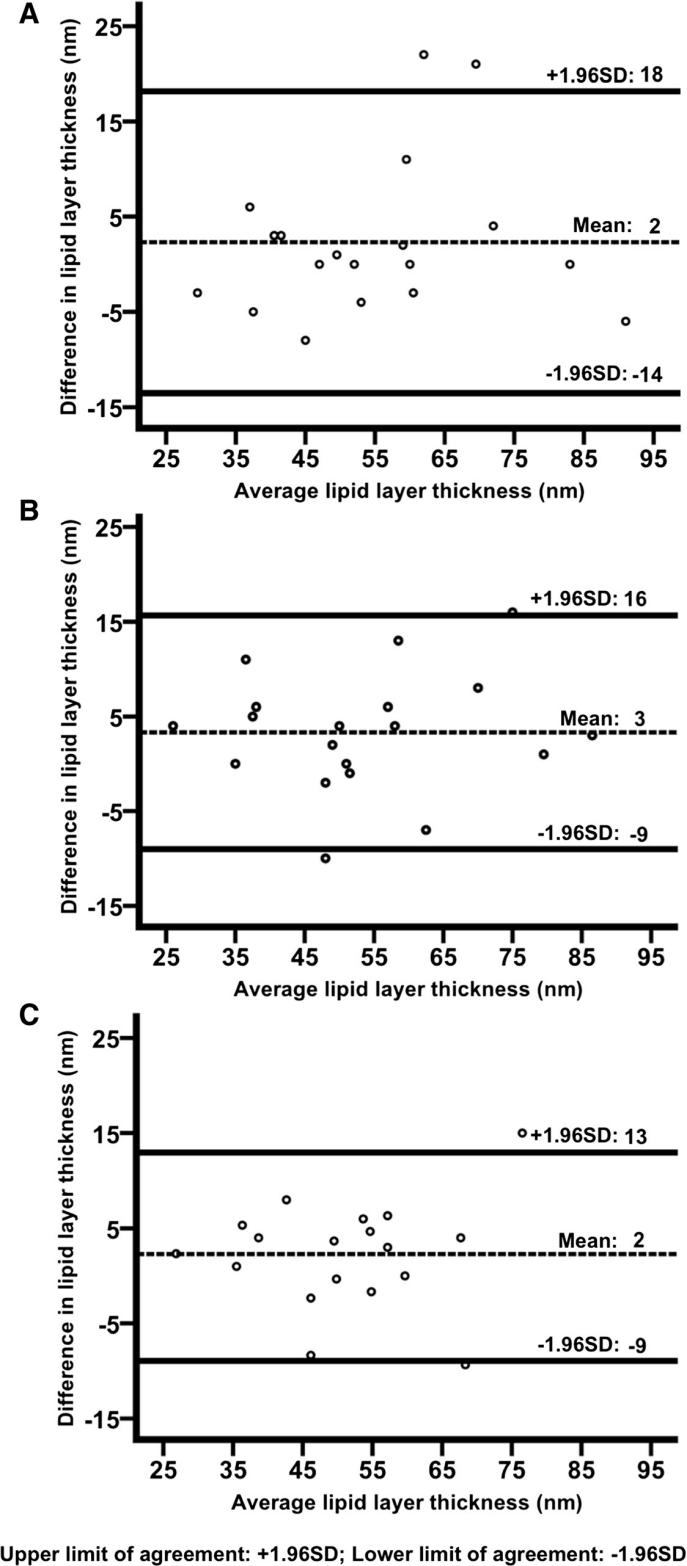
The Bland Altman plots for intra and inter-observer assessment of LLT using the interferometer. The difference between 2 measurements was plotted against the average of the measurements (nm). For the randomly selected eyes, the intra-observer repeatability (**a**), single scan, inter-observer repeatability (**b**) and averaged-3-scan inter-observer repeatability (**c**) plots are shown. +95 %SD and −95 %SD refer to the upper limit and lower limit of agreement respectively

In terms of intra-observer repeatability, the Bland-Altman plot showed a coefficient of repeatability of 16 nm and limits of agreement (95 % CI of differences) between −14 nm and 18 nm (Fig. 1a).

For inter-observer repeatability, when a single scan of one observer was compared to that of the other observer, the coefficient of repeatability was 13 nm. In the Bland-Altman plot (Fig. 1b), the limits of agreement were between −9 nm and 16 nm. When the averaged triplicate measurement of one observer was compared to that of the other, the coefficient of repeatability was 11 nm. Its corresponding limits of agreement (Fig. 1c) were −9 nm and 13 nm.

There was no significant systematic error in the intra-observer and inter-observer comparisons, with a mean difference of 2 nm and 3 nm respectively. In all the analyses above, the differences were not associated with the magnitude of the means. 1–2 outliers were removed from the Bland-Altman plots before analysis.

### Possible associations and correlation with clinical parameters

The median TBUT (minimum, maximum) was 3 (2, 7) s and 70 % of the participants had a TBUT of less than 5 s (Table 1). TBUT was not significantly correlated with LLT (p = 0.874, Spearman’s r = 0.038).

Among the participants, 40 % had corneal fluorescein staining in at least one of the 5 corneal zones (Table 1). LLT was not significantly associated with the presence of any corneal staining (p = 0.325). In addition, LLT was not significantly associated with age, gender, history of current contact lens wear, history of ocular surgery and symptomatic status for dry eyes on univariate and multivariate analysis (p > 0.05).

### Assessment after scanning

Amongst the participants, 60 % had visual acuity of 6/9 or better, while 15 % were from 6/9^−1^ to 6/12 and 25 % were from 6/12^−1^ to 6/18. None of the participants who went through interferometry complained about increased discomfort.

## Discussion

For any given reading of the same subject, LLT was found not to differ from the mean by more than 16 nm. The limits of agreement for inter-observer and intra-observer measurements were similar. This suggests that the repeatability of measurements was independent of its observer. The single-scan inter-observer agreement and the average of triple-scan inter-observer LLT measurements were also similar. There was no systematic difference between different measurements (whether intra-observer or inter-observer) as mean differences were not significantly different from zero. In this study, none of the tested clinical factors were associated with LLT. Despite the high number of scan acquisitions, no participant complained of discomfort.

Prior study on the repeatability of quantitative LLT measurements using interferometry involved a new spectral interferometer that has been developed by Fogt and King-Smith [12, 30, 47]. However, this interferometer is not commercially available. Moreover, although the study reported a good correlation coefficient (Spearman’s r = 0.835) [17], it must be noted that correlation alone is not an appropriate measure of repeatability [46].

Repeatability was also assessed in the pre-production model of the LipiView® interferometer. The pre-production model measured LLT by asking two observers to determine the LLT based on subjective appreciation of the interference colors and these measurements were found not to defer by more than 30 nm [9]. Compared to its pre-production model, the commercially released interferometer employed software processing and analysis of the recorded interference colors to calculate LLT [10]. As such, repeatability of measurements may be improved by the objective nature of software analysis.

To put our repeatability finding in perspective, mean LLT (SD) was 76 (25) nm in dry eye patients in a study by Finis et al. [16], and 65.0 (19.1) nm and 54.2 (17.9) nm respectively in healthy controls and MGD patients in another study by Eom et al. [6]. In our Asian study population, the average LLT of 54 (15) nm appeared to be lower than these two studies. The difference may be due to greater heterogeneity in our study sample.

In terms of correlations between LLT and TBUT or fluorescein staining, this study showed no significant correlations, and was generally similar with these two prior studies [6] [16]. In Finis et al’s study, a weak correlation between LLT and Ocular Surface Disease Index (OSDI) (r = −0.13) was found, whilst we did not find any difference in LLT between symptomatic and non-symptomatic participants. In Eom et al’s study, correlation between LLT and TBUT was only found in the MGD group (r = 0.415). The primary objective of our study was to investigate repeatability and not to examine associations with clinical parameters of dry eye or meibomian gland dysfunction. Inter-ethnic differences, if any, will require future studies to elicit.

The strength of our study is that it was conducted in a very controlled condition with trained observers. Our study had the following limitations. Firstly, during LLT measurement, actual room humidity and temperature were not actually measured. However, the evaluation room was centrally air-conditioned and Singapore does not have any seasonal variations in climate. Secondly, as the sample size was small, differences in repeatability of measurements in different patient subgroups could not be assessed. Thirdly, the repeatability results could not be generalized to patients with specific characteristics which differ from this study’s sample.

In the future, technological advances may improve the repeatability of the instrument further. In software analysis, perhaps the pattern (open meshwork, closed meshwork, wave, colour fringe) can be considered in addition to the colour of the spectral reflection. Statistical modeling with these additional variables may more precisely estimate LLT measurements. It may also be desirable for interferometry to be able to function over a wider area of the cornea or in a specific area of the cornea, or perhaps to measure LLT in non-blinking (stressed) conditions. A Kowa DR-1-based software has been developed to sample LLT over multiple corneal zones and interpret kinetic changes in tear lipid spreading [32, 37, 48].

Now that a certain degree of repeatability is established, interferometry may be a useful modality in monitoring changes of LLT in clinical trials. Single scan on each occasion is adequate for LLT measurement, and repeat measurements need not be performed by the same examiner.

## Conclusion

With the repeatability of measurements being known, the significance of treatment-induced changes in the LLT measurements of this interferometer may be better interpreted. This is useful in clinical studies where a group of patients has undergone intervention related to the tear film. The size of the group will need to be determined by the treatment effect that these future studies aim to detect.

## Abbreviations

LLT: Lipid layer thickness
MGD: Meibomian gland dysfunction
TBUT: Tear break-up time
CCLRU: Cornea and Contact Lens Research Unit
SD/s.d.: Standard deviation
95 %CI: 95 % Confidence interval
